# The Enhancing Effect of Stable Oxygen Functional Groups on Porous-Carbon-Supported Pt Catalysts for Alkaline Hydrogen Evolution

**DOI:** 10.3390/nano13081415

**Published:** 2023-04-20

**Authors:** Xianyou Luo, Ping Yuan, Junhui Luo, Haoming Xiao, Junyi Li, Heng Zheng, Baodong Du, De Li, Yong Chen

**Affiliations:** 1 State Key Laboratory of Marine Resource Utilization in South China Sea, Hainan Provincial Key Laboratory of Research on Utilization of Si-Zr-Ti Resources, Hainan University, Haikou 570228, China; 2 Guangdong Key Laboratory for Hydrogen Energy Technologies, School of Materials Science and Hydrogen Energy, Foshan University, Foshan 528000, China

**Keywords:** oxygen functional groups, porous carbon, support, hydrogen evolution reaction, Pt/C catalysts

## Abstract

**Highlights:**

**What are the main findings?**

- The HCl treatment of porous carbon can generate abundant hydroxyl and carboxyl groups, while the further heat treatment can transform into thermally stable carbonyl and ether groups.
- Carbonyl and ether groups within porous carbon supports is beneficial to the improvement of HER performance of catalysts.

**What is the implication of the main finding?**

- The surface properties of porous carbon supports can be well tuned via a HCl treatment followed by an appropriate heat treatment.
- The feasible improvement of HER performance by regulating surface oxygen functional groups of porous carbon supports.

**Abstract:**

The oxygen functionalization of carbon materials has widely been employed to improve the catalytic performance of carbon-supported Pt (Pt/C) catalysts. Hydrochloric acid (HCl) has often been employed to clean carbons during the preparation of carbon materials. However, the effect of oxygen functionalization through a HCl treatment of porous carbon (PC) supports on the performance of the alkaline hydrogen evolution reaction (HER) has rarely been investigated. Herein, the impact of HCl combined with the heat treatment of PC supports on the HER performance of Pt/C catalysts has been comprehensively investigated. The structural characterizations revealed similar structures of pristine and modified PC. Nevertheless, the HCl treatment resulted in abundant hydroxyl and carboxyl groups and the further heat treatment formed thermally stable carbonyl and ether groups. Among the catalysts, Pt loading on the HCl-treated PC followed by a heat treatment at 700 °C (Pt/PC-H-700) exhibited elevated HER activity with a lower overpotential of 50 mV at 10 mA cm^−2^ when compared to the unmodified Pt/PC (89 mV). Pt/PC-H-700 also exhibited better durability than the Pt/PC. Overall, novel insights into the impact of the surface chemistry properties of porous carbon supports on the HER performance of Pt/C catalysts were provided, which were useful for highlighting the feasible improvement of HER performances by regulating the surface oxygen species of porous carbon supports.

## 1. Introduction

Hydrogen is not only useful as a significant energy carrier with high-energy density and environmentally friendly aspects, but also as an intermediate in the synthesis of high-value compounds [1,2,3]. Most hydrogen is currently produced from steam-reformed methane generated by limited fossil resources, resulting in an enormously increased CO_2_ emission [4]. This issue can be solved by producing hydrogen through the electrocatalytic hydrogen evolution reaction (HER) from water splitting. This process is efficient and advantageous in terms of its low-cost, safe, and large-scale pathway for H_2_ production [5,6,7].

Platinum (Pt) is the most efficient electrocatalyst for the HER, owing to its favorable Gibbs adsorption-free energy of H* (∆G_H*_) [5,8,9]. However, the natural scarcity and high cost of Pt largely limit its large-scale applications. Alternatively, Pt-supported catalysts are advantageous in terms of their high active surface areas, increased utilizations, and strong metal–support interactions [10,11,12,13]. Among these supports, carbon-based materials have widely been employed due to their large specific surface areas, high electrical conductivities, good physicochemical stabilities, and adjustable surface chemistry properties [14,15]. These include carbon nanofibers, carbon nanotubes, ordered mesoporous carbons, and carbon blacks [6], which have widely been explored as the supports of Pt-based catalysts (Pt/C) [11,16,17,18]. Generally, the catalytic efficiency of carbon-supported Pt catalysts is significantly associated with both the used carbon support and the supported Pt [6]. In this respect, an N-doped mesoporous hollow carbon-spheres-supported Pt single atoms catalyst (Pt_1_/NMHCS) is much more active and stable than the commercial 20% Pt/C and Pt nanoparticle (Pt_NP_) counterparts for the HER [10]. This catalyst can deliver a low overpotential of 40 mV at 10 mA cm^−2^, a high mass activity of 2.07 A mg^−1^_Pt_ at a 50 mV overpotential, a low Tafel slope of 56 mV dec^−1^, and an excellent durability in acidic electrolyte. Owing to the high dispersion of Pt, its hollow structure, large surface area, and nitrogen dopant and hierarchical pore features, a nitrogen-doped hollow porous carbon polyhedrons-supported dispersed Pt nanoparticles (Pt/NHPCP) catalyst exhibited an enhanced HER activity and durability when compared to commercial Pt/C [19]. The Pt/NHPCP catalyst exhibited excellent HER activity, with an overpotential of 57 mV at 10 mA cm^−2^ and a Tafel slope of 27 mV dec^−1^, much lower than that of commercial Pt/C (69.9 mV and 32 mV dec^−1^). Similarly, a functionalized multiwall carbon-nanotubes-supported Pt single-atom and cluster (Pt/f-MWCNTs) catalyst exhibited outstanding mass activity and stability for catalyzing the HER in an acid medium, due to the unique surface properties of its carbon surface [20]. Owing to its unique active moiety (Pt_1_-O_2_-Fe_1_-N_4_), the Pt_1_@Fe-N-C electrocatalyst exhibited a high catalytic activity toward the HER with a low overpotential of 60 mV at 10 mA cm^−2^ and a small Tafel slope of 42 mV dec^−1^ in acidic media [21].

In general, the structural and surface properties of carbon supports can significantly influence the states of supported Pt species, thereby regulating the catalytic performance of Pt/C catalysts. Chemical treatment can regulate the surface chemistry properties of these carbon supports, especially their oxygen functionalization, which can improve the catalytic performance of the Pt/C catalysts. For instance, the surface properties of carbons can be changed by HNO_3_, H_2_SO_4_, KOH, H_3_PO_4_, HF, C_6_H_6_, and H_2_O_2_ treatments [16,18,22,23,24,25]. The oxygen functionalization of carbon materials via HNO_3_ and H_2_O_2_ may introduce abundant surface oxygen functional groups on carbon supports to disperse/anchor their Pt nanoparticles and enhance their metal–support interactions, thereby improving the catalytic performances of the catalysts. However, different oxidation reagents often result in different electrochemical performance electrocatalysts. For example, Pt/C catalysts synthesized by the H_2_O_2_ treatment of carbon supports have exhibited a higher cycling stability than those prepared by a HNO_3_ treatment [22]. KOH-treated carbon black (CB)-supported Pt catalysts have shown better electrocatalytic activities than C_6_H_6_- or H_3_PO_4_-treated CB-supported Pt catalysts [23]. HNO_3_-treated carbon-black-supported Pt catalysts have depicted higher electrochemically active surface areas, better oxygen reduction reaction activities, and lower stabilities than unmodified carbon-black-supported Pt catalysts [16]. Therefore, investigating the effects of oxygen functional groups on the HER performances of Pt/C catalysts is vital, due to the diversity of the oxygen functional groups on the carbon surface and their complex impacts on the electrocatalytic performances. On the other hand, since hydrochloric acid (HCl) is often utilized to remove impurities during the preparation of carbon materials [26,27,28,29,30], carbon materials washed by HCl might have different surface chemical properties, but are usually neglected during studies. Therefore, investigating the effects of a HCl treatment of carbon supports on the HER performances of Pt/C catalysts is of great significance for the catalysis field and energy applications.

Herein, porous carbon (PC) derived from coconut shell was utilized as a support for preparing a series of Pt/C catalysts. The effects of the HCl treatment coupled with a further heat treatment on the HER performances of Pt/C catalysts were elaborately investigated. The structural characterizations revealed the similar porous structures of the pristine PC and modified PC. However, the HCl treatment induced abundant oxygen functional groups, such as hydroxyl and carboxyl groups, while the subsequent heat treatment formed thermally stable oxygen groups such as carbonyl and ether groups. Among the prepared samples, the optimized Pt/PC-H-700 displayed an improved HER catalytic activity, with a lower overpotential of 50 mV at 10 mA cm^−2^ when compared to the unmodified Pt/PC (89 mV). Meanwhile, the catalytic stability of the Pt/PC-H-700 was better than that of the Pt/PC from the durability measurements.

## 2. Experimental

### 2.1. HCl Treatment of PC

The PC used in this work was derived from coconut shell and prepared by the water–gas activation method. Typically, the coconut shell was first directly carbonized at 450 °C for 1 h and then activated by water steam at 880 °C for 2.5 h, at a heating rate of 10 °C min^−1^. In this reaction, the mass ratio of steam to carbon was fixed at 3:1. Afterward, the PC (10 g) and hydrochloric acid (HCl, 10 vol%, 100 mL) were added into a round-bottomed glass flask, followed by mounting in an oil bath device and boiling (100 °C) for 5 h. The obtained product after the HCl treatment was taken out, cooled, washed with deionized water until it reached a neutral pH, and dried in a vacuum oven at 60 °C for 12 h to yield the PC-H samples.

### 2.2. Heat Treatment of PC-H

The annealing treatment of the PC-H was performed under an Ar atmosphere at a flow rate of 50 mL min^−1^. Briefly, the as-obtained PC-H (1 g) was placed into a tube furnace and heated at a rate of 5 °C min^−1^ from room temperature to the target temperature (500 °C, 600 °C, 700 °C, and 800 °C) for 2 h, before being cooled down to room temperature to yield PC-H-500, PC-H-600, PC-H-700, and PC-H-800, respectively.

### 2.3. Preparation of Pt/C Catalysts

The Pt/C catalyst was obtained by a modified polyol reduction method, with the Pt loading content fixed at 5 wt.% [31,32]. Typically, the PC (50 mg) was first suspended in an ethylene glycol solution and subsequently stirred in an ultrasonic bath for 10 min. A chloroplatinic acid hexahydrate (H_2_PtCl_6_·6H_2_O, 39 µL) solution at a Pt concentration of 63.885 mg mL^−1^ was then slowly added dropwise and stirred for 30 min before heating at 140 °C for 3 h in an oil bath. After being cooled down to room temperature, the prepared catalyst was taken out and washed with ethanol and deionized water until it reached a neutral pH, followed by drying at 60 °C for 12 h to yield the Pt/PC catalyst. The other catalysts, such as Pt/PC-H, Pt/PC-H-500, Pt/PC-H-600, Pt/PC-H-700, and Pt/PC-H-800, were obtained through the same preparation process using PC-H, PC-H-500, PC-H-600, PC-H-700, and PC-H-800 as supports, respectively.

### 2.4. Structural Characterization

The surface morphologies and elemental distributions of the carbon supports were studied by scanning electron microscopy (SEM, Phenom ProX) equipped with energy dispersive spectroscopy (EDS). The sizes and distributions of the Pt nanoparticles were visualized by transmission electron microscopy (TEM, FEI-Tecnai G2 F20). The crystalline structures of the samples were identified by X-ray diffraction (XRD, Bruker D8 Advance) equipped with monochromated Cu Kα1 radiation (λ = 1.5418 Å). The defect information of each porous carbon was obtained by Raman spectroscopy (Thermo Fisher DXRxi) equipped with a laser source of 532 nm. The surface oxygen functional groups of the carbons were examined by Fourier transform infrared spectroscopy (FTIR, PerkinElmer frontier instrument). The surface chemical compositions of the samples were identified by X-ray photoelectron spectroscopy (XPS, Thermo Scientific Nexsa) equipped with an Al anode (Al Kα = 1486.68 eV). The Ar adsorption–desorption isotherms of the carbon supports were obtained on a Quantachrome surface area analyzer (Autsorb IQ-2) at 87 K. The specific surface areas were calculated by the Brunauer–Emmett–Teller (BET) equation and the pore size distributions were determined by the quenched solid density functional theory (QSDFT), based on a slit-pore model.

### 2.5. Electrochemical Measurements

The electrocatalytic performances were tested on an electrochemical workstation (Ivium) connected to a typical three-electrode cell filled with 1 M KOH electrolyte at room temperature. An Ag/AgCl electrode (3.5 M KCl) and graphite electrode were employed as the reference and counter electrodes, respectively. The working electrodes consisted of an L-shaped glassy carbon (GC) substrate with a diameter of 5 mm and a geometric area of 0.196 cm^−2^. The working electrodes were obtained by carefully dropping 10 µL of homologous catalyst ink, which was prepared by mixing 3 mg of the catalyst and 50 µL of a 5 wt% Nafion solution in 450 µL of anhydrous ethanol, sonicated for 0.5 h on a GC surface and followed by drying in air.

The HER catalytic activity was studied by linear sweep voltammetry (LSV) at a scan rate of 2 mV s^−1^. The reaction kinetics were examined by electrochemical impedance spectroscopy (EIS) at an overpotential of 90 mV and amplitude of 5 mV (10^5^ Hz–0.1 Hz). The accelerated durability test (ADT) was evaluated using 5000 cyclic voltammetry (CV) profiles from −0.8 V to −1.3 V at 100 mV s^−1^. Long-term chronopotentiometry (V-t) measurements were conducted at 10 mA cm^−2^ for 10 h. Time-dependent current density (I-t) profiles were collected at a fixed overpotential of 110 mV.

## 3. Results and Discussion

**Figure 1:**
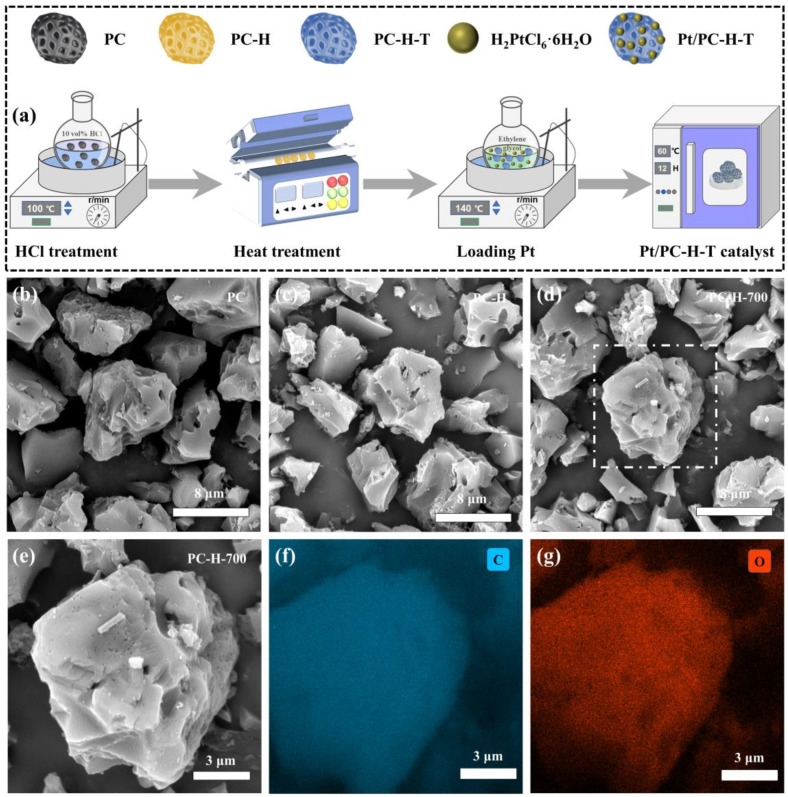
(**a**) Scheme representing the synthesis process of Pt/PC-H-T catalyst. SEM images of PC (**b**), PC-H (**c**), and PC-H-700 (**d**) supports. High-magnification SEM images and corresponding EDS mappings of PC-H-700 (**e**–**g**) support.

The synthesis process of the Pt/PC-H-T catalyst is schematically illustrated in Figure 1a. Briefly, the PC-H was achieved by the HCl (10 vol%) treatment of the PC at 100 °C for 5 h. The further heat treatment of the PC-H at the target temperature for 2 h yielded the PC-H-T sample. Finally, the Pt/PC-H-T catalyst was synthesized by the solvothermal reduction method at 140 °C for 3 h. 

The structural characteristics of the as-prepared supports were investigated by various analytical methods. The SEM morphologies of the PC, PC-H, and PC-H-700 supports are depicted in Figure 1b–d. Obviously, the PC, PC-H, and PC-H-700 exhibited similar irregular particles and porous structures, which were conducive to providing abundant anchoring sites for the Pt species. Additionally, the PC-H and PC-H-700 still maintained the pristine PC morphology after the HCl treatment and heat treatment. The high-magnification SEM images (Figure 1e–g) and corresponding EDS mappings of the PC-H-700 (white dashed box in Figure 1d) revealed uniform distributions of C and O elements. 

**Figure 2:**
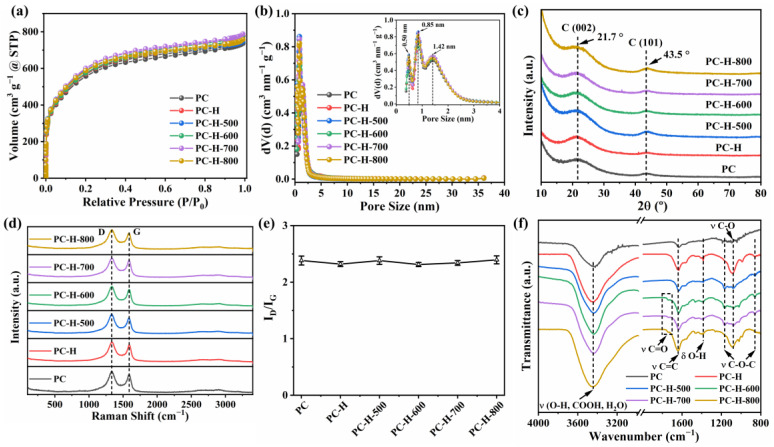
(**a**) Ar adsorption–desorption isotherms, (**b**) pore size distribution curves, (**c**) XRD patterns, (**d**) Raman spectra, (**e**) I_D_/I_G_ ratios, and (**f**) FTIR spectra of PC, PC-H, PC-H-500, PC-H-600, PC-H-700, and PC-H-800 supports.

The porosity of each support was examined with the Ar adsorption–desorption isotherms and the results of PC, PC-H, PC-H-500, PC-H-600, PC-H-700, and PC-H-800 are provided in Figure 2a. All the carbon supports exhibited similar Ar adsorption–desorption isotherm shapes (combined I/IV-type), with typical H4-type hysteresis loops [33]. The rapid upward trend in the low Ar pressure (P/P_0_ < 0.01) suggested the presence of abundant micropores in all the supports. The continuous rise in the high P/P_0_ range and the existence of H4-type hysteresis loops (0.4 < P/P_0_ < 0.98) demonstrated the presence of mesopores due to the capillary condensation phenomenon of the liquid Ar in mesopores. These results confirmed the formation of a hierarchical porous structure in all the carbon supports, which was conducive to providing abundant active sites for Pt dispersion and anchoring, as well as offering adequate catalyst/electrolyte interfaces for charge/ion accumulation. The QSDFT pore size distributions of all the carbon supports are displayed in Figure 2b. All the samples showed similar pore size distribution curves, mainly centered around 0.4–4 nm, which is consistent with the above Ar adsorption–desorption isotherms analysis. The dominant pore sizes in the PC, PC-H, PC-H-500, PC-H-600, PC-H-700, and PC-H-800 supports were identified as 0.50 nm, 0.85 nm, and 1.42 nm (inset of Figure 2b). Further details related to the pore structure parameters, including the specific surface areas and pore volumes, are summarized in Table 1 and Figure S1 of the Supporting Information.

The crystal structures of the carbon supports were examined by XRD. As shown in Figure 2c, all the samples displayed two similar broad diffraction peaks at around 21.7° and 43.5°, corresponding to the (002) and (100) crystalline planes of carbon, respectively. Thus, all the carbon supports showed amorphous features [34,35,36], which were further confirmed by Raman spectroscopy. As depicted in Figure 2d, all the carbon supports demonstrated two representative peaks at ~1330 cm^−1^ and ~1589 cm^−1^, which were ascribed to the D-band (A_1g_ breathing-mode vibration of C_6_ rings, K-point phonons of A_1g_ symmetry) and G-band (E_2g_ vibration of sp^2^-bonded carbon pairs, zone center phonons of E_2g_ symmetry), respectively [7,37,38]. In Figure S2, the Raman spectra of the carbon supports were further fitted into four Lorentz peaks, which were located at ~1215, ~1335, ~1525, and ~1590 cm^−1^, corresponding to the I-, D-, D″-, and G-bands, respectively. In general, the intensity ratio between the D-band and G-band (I_D_/I_G_) can be utilized to quantify the defect densities or graphitization degrees of carbon materials [39,40]. Based on the peak areas, the I_D_/I_G_ values of the PC, PC-H, PC-H-500, PC-H-600, PC-H-700, and PC-H-800 supports were determined to be 2.38, 2.32, 2.38, 2.31, 2.34, and 2.39, respectively. Therefore, the HCl treatment followed by the heat treatment did not drastically change the defect density of the PC (Figure 2e).

The surface oxygen functional groups of the carbon supports were examined by an FTIR analysis. As described in Figure 2f, all the FTIR spectra showed a broad and intense absorption peak at ~3438 cm^−1^, which was assigned to the stretching vibration of the O-H (ν O-H) in the hydroxyl groups, carboxyl groups, or adsorbed water [41,42]. The typical peak centered at ~1634 cm^−1^ belonged to the C=C skeletal vibration of the aromatic rings (ν C=C) [43,44]. To identify the oxygen functional groups, the fingerprint band ranging from 1600 to 800 cm^−1^ was carefully investigated. For the unmodified PC, the characteristic band of the oxygen functional groups looked very faint. The peak centered at 1385 cm^−1^ can be attributed to the bending vibrations of the O-H (δ O-H) [29,45]. The broad band from 1250 cm^−1^ to 900 cm^−1^ may be ascribed to the C-O vibration. After the HCl treatment, the peak intensity of the PC-H at ~1089 cm^−1^ (ν C-O) obviously increased, suggesting enhanced oxygen functional groups on the PC surface. The further heat treatment of the PC-H resulted in the emergence of two peaks located at ~1168 cm^−1^ and ~1078 cm^−1^, which were attributed to the stretching vibration of C-O-C (ether) and C-O (ν C-O), respectively [43,46]. A peak around ~857 cm^−1^ (ν C-O-C) appeared in the PC, PC-H-500, PC-H-600, PC-H-700, and PC-H-800 [47]. Another new peak at ~1736 cm^−1^ also emerged at heating temperatures of over 600 °C, which was ascribed to the stretching vibration of C=O (ν C=O) [16,25,48]. Therefore, the HCl treatment followed by the heat treatment formed thermally stable C-O-C and C=O species [43,44].

**Figure 3:**
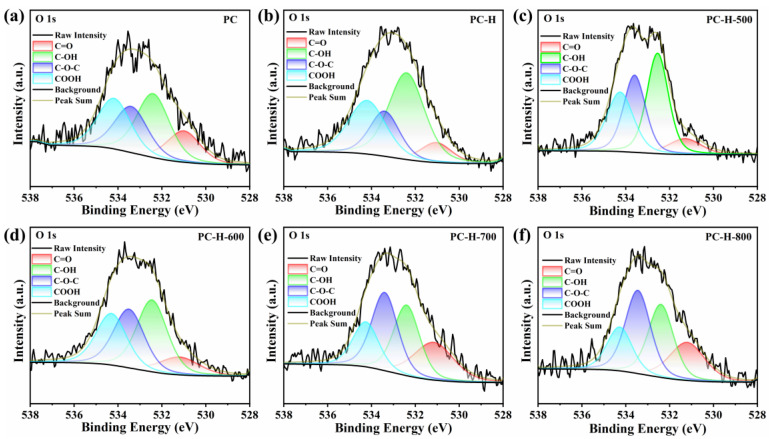
High-resolution O 1s spectra of PC (**a**), PC-H (**b**), PC-H-500 (**c**), PC-H-600 (**d**), PC-H-700 (**e**), and PC-H-800 (**f**) supports.

**Figure 4:**
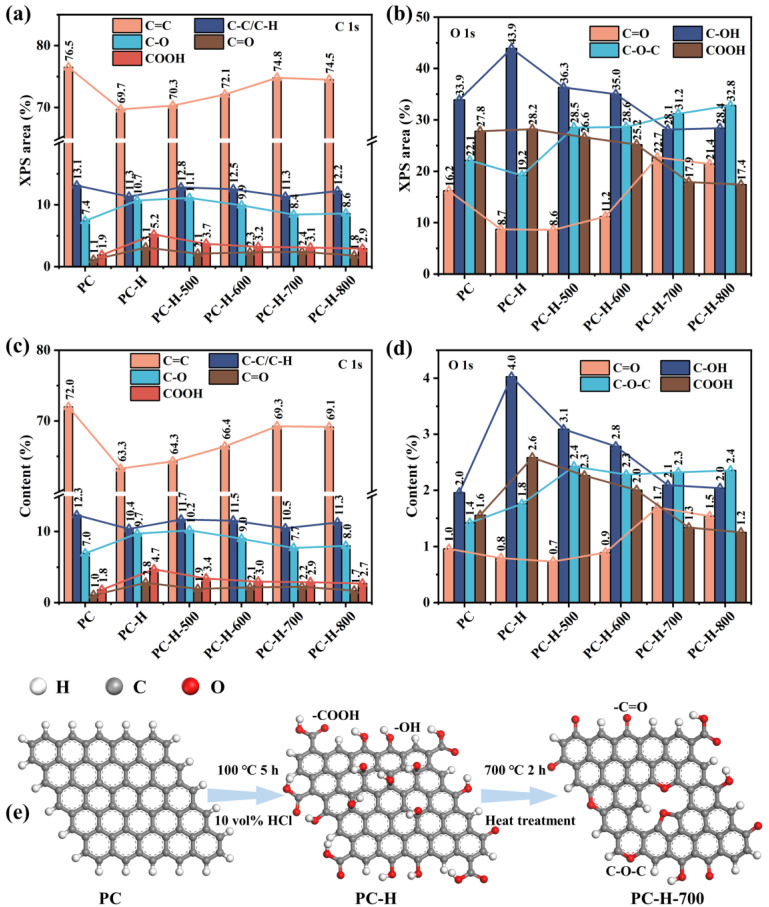
Histograms of XPS area ratios deconvoluted from C 1s (**a**), and O 1s (**b**). Histograms of the content ratios deconvoluted from C 1s (**c**), and O 1s (**d**). (**e**) Schematic representation of oxygen functional groups on modified PC.

The surface chemistry of the carbon supports was investigated by XPS. As illustrated in Figure S3, the survey spectra of all the carbon supports exhibited the presence of C and O elements. The samples of the PC, PC-H, PC-H-500, PC-H-600, PC-H-700, and PC-H-800 all contained O heteroatom at atomic percentages of 5.89%, 9.16%, 8.51%, 7.96%, 7.44%, and 7.18%, respectively. Thus, the O content in each carbon support increased after the HCl treatment, while declining after the heat treatment. The high-resolution C 1s spectra could be deconvoluted into five peaks at 284.8 eV, 285.6 eV, 286.4 eV, 287.6 eV, and 289.5 eV, corresponding to C=C, C-C/C-H, C-O, C=O, and COOH, respectively (Figure S4) [49,50,51,52]. As illustrated in Figure 3, the high-resolution O 1s spectra could be fitted with four major peaks at 531.2 eV, 532.3 eV, 533.4 eV, and 534.3 eV, representing C=O (carbonyl), C-OH (hydroxyl), C-O-C (ether), and COOH (carboxyl), respectively [44,53,54]. In Figure 4a,c, the sp^2^ C=C content of the PC decreased after the HCl treatment, leading to rising trends of the C-O, C=O, and COOH contents. The subsequent heat treatment of the PC resulted in a gradual enhancement of the sp^2^ C=C content, while declining the C-O, C=O, and COOH contents. A further analysis of the high-resolution O 1s deconvolution spectra revealed more intuitive components of the oxygen functional groups. In Figure 4b,d, the PC-H displayed higher C-OH and COOH contents than the unmodified PC after the HCl treatment. As the heat treatment’s temperature rose, the C-OH and COOH contents gradually decreased, while the C=O and C-O-C contents increased, demonstrating the benefit of the heat treatment in the formation of thermally stable C=O (carbonyl) and C-O-C (ether) groups [44,45,55], which is consistent with the above FTIR analysis. Accordingly, a scheme representing the formation of thermally stable oxygen functional groups on the modified PC was drawn and can be seen in Figure 4e.

**Figure 5:**
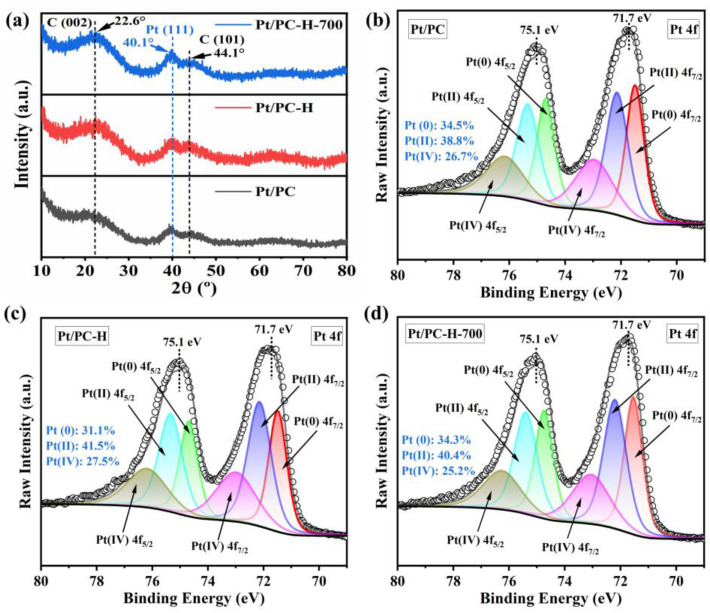
XRD patterns (**a**) and high-resolution Pt 4f spectra of Pt/PC (**b**), Pt/PC-H (**c**), and Pt/PC-H-700 (**d**) catalysts.

After loading the Pt on the carbon supports, the existential states of the Pt in the Pt/PC, Pt/PC-H, and Pt/PC-H-700 catalysts were studied by XRD. As shown in Figure 5a, two obvious broad diffraction peaks were noticed at 22.6° and 44.1°, corresponding to the (002) and (101) planes of graphitic carbon. A slight intensity Pt diffraction peak was also observed at a 2θ value of 40.1°, corresponding to the (111) crystal plane of the Pt (JCPDS No. 04-0802). Thus, the Pt nanoparticles in the Pt/PC, Pt/PC-H, and Pt/PC-H-700 catalysts mainly exposed the Pt (111) crystal plane. The surface chemistry states of the Pt/PC, Pt/PC-H, and Pt/PC-H-700 catalysts were studied by XPS. As displayed in Figure S5, the survey spectra of the catalysts showed the presence of C, O, and Pt elements, indicating that the Pt species was successfully loaded on the carbon supports. The high-resolution C 1s and O1s spectra of the Pt/PC, Pt/PC-H, and Pt/PC-H-700 catalysts were further deconvoluted to investigate the contents of the components. These detailed deconvolution results are depicted in Figure S6 and Tables S1 and S2. The chemical states of the Pt in the catalysts were studied by deconvoluting the high-resolution Pt 4f XPS spectra (Figure 5b–d). The Pt 4f XPS spectra of the Pt/PC, Pt/PC-H, and Pt/PC-H-700 catalysts displayed doublet peaks at about 71.7 eV and 75.1 eV, which could further be deconvoluted into three peaks. The deconvoluted doublet peaks at (71.5 eV and 74.7 eV), (72.2 eV and 75.4 eV), and (73 eV and 76.2 eV) were assigned to (Pt^0^ 4f_7/2_ and Pt^0^ 4f_5/2_), (Pt^2+^ 4f_7/2_ and Pt^2+^ 4f_5/2_), and (Pt^4+^ 4f_7/2_ and Pt^4+^ 4f_5/2_), respectively [10,12,16]. The quantification of the integrated areas of these deconvoluted peaks in the Pt 4f spectra (Table S3) revealed the presence of oxidized Pt^δ+^ (such as Pt^2+^ and Pt^4+^) species, accounting for 65–69% of the total number of the Pt nanoparticles. This could be attributed to the electron transfer process from the Pt to the carbon supports, thanks to the higher electronegativity of the C and O when compared to the Pt. On the other hand, the oxophilic microscopic Pt nanoparticles formed a dissolution-inhibiting passivation layer of PtO_x_, which was useful for increasing the number of Pt^δ+^ species and improving the catalyst’s stability [56]. The SEM images of the Pt/PC-H-700 catalyst are displayed in Figure S7a,b. Obviously, the Pt/PC-H-700 catalyst still exhibited similar irregular particles and porous structures to the PC-H-700 support. In addition, the EDS mappings of the Pt/PC-H-700 catalyst also revealed a uniform distribution of the C, O, and Pt elements (Figure S7c–f), which indicates that the Pt species were successfully deposited onto the carbon support.

**Figure 6:**
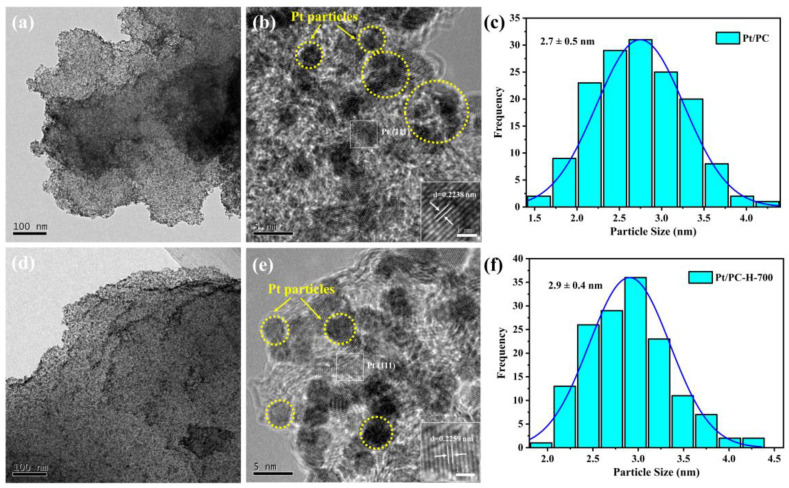
TEM and HRTEM images of Pt/PC (**a**,**b**), and Pt/PC-H-700 (**d**,**e**) catalysts. Pt size distributions of Pt/PC (**c**), and Pt/PC-H-700 (**f**) catalysts.

The distributions and sizes of the Pt nanoparticles were visualized by TEM/HRTEM and the results of the Pt/PC and Pt/PC-H-700 catalysts are gathered in Figure 6. Compared to the Pt/PC catalyst (Figure 6a,b), the Pt nanoparticles in the Pt/PC-H-700 catalyst (Figure 6d,e) looked more uniformly dispersed on the carbon support, while those of the Pt/PC catalyst underwent a slight agglomeration. Meanwhile, the Pt nanoparticles (yellow dotted line circle) are clearly observed in Figure 6b,e. In addition, the Pt/PC-H-700 catalyst displayed a larger Pt (111) lattice spacing of 0.2259 nm than that of the Pt/PC catalyst (0.2238 nm), indicating the presence of abundant thermally stable C=O and C-O-C groups on the carbon support, which were suitable for generating tensile strain on the Pt nanoparticles, consistent with previous research [57].

The Pt particle size distribution was acquired by measuring the average sizes of the 150 Pt nanoparticles from the TEM images of the Pt/PC and Pt/PC-H-700 catalysts (Figure S8). The Pt/PC catalyst exhibited an average Pt nanoparticle size of ~2.7 nm, a value that was slightly smaller than that of the Pt/PC-H-700 catalyst (~2.9 nm) (Figure 6c,f). However, the more uniform Pt nanoparticle distribution of the Pt/PC-H-700 catalyst may deliver better HER catalytic performances than those of the Pt/PC catalyst. In general, surface oxygen functional groups would play two functions during the supported catalyst preparation. The first would consist of decreasing the hydrophobic character of the carbon support via the surface oxygen functional groups [58]. The other would rely on them acting as Pt anchoring sites, thereby hindering the redistribution and agglomeration of the Pt nanoparticles during the Pt precursor reduction stage [25]. This effect is consistent with the previous research results for the use of carbon-based catalysts [59,60]. Therefore, the presence of enriched thermally stable C=O and C-O-C groups on carbon supports would accommodate abundant active sites for Pt species. 

**Figure 7:**
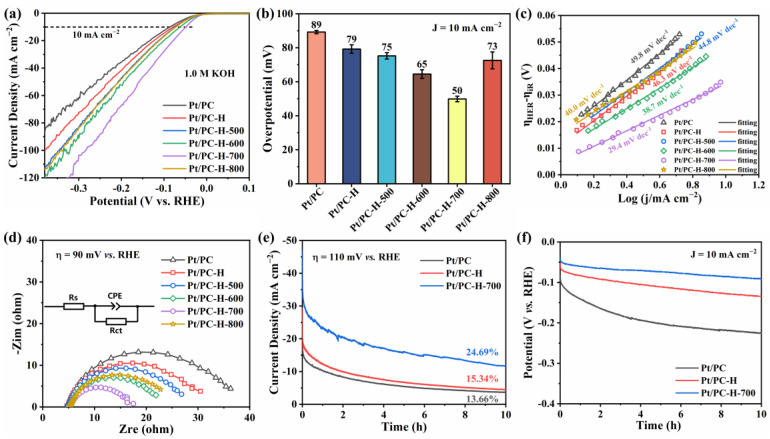
(**a**) LSV curves, (**b**) overpotential at 10 mA cm^−2^, (**c**) Tafel slope values, and (**d**) EIS plots (inset shows the equivalent circuit model) of Pt/PC, Pt/PC-H, Pt/PC-H-500, Pt/PC-H-600, Pt/PC-H-700, and Pt/PC-H-800 catalysts. I-t (**e**) and V-t (**f**) curves of Pt/PC, Pt/PC-H, and Pt/PC-H-700 catalysts.

The HER performances of the as-prepared catalysts were investigated by evaluating the HER electrocatalytic activities of the Pt/PC, Pt/PC-H, Pt/PC-H-500, Pt/PC-H-600, Pt/PC-H-700, and Pt/PC-H-800 catalysts by their LSV measurements. As displayed in Figure 7a,b, compared to the Pt/PC catalyst, significantly improved HER performances were observed for all the modified PC-supported Pt catalysts. In particular, the optimized Pt/PC-H-700 catalyst revealed a superior HER catalytic activity with a low overpotential of 50 mV at a current density of 10 mA cm^−2^, values lower than those of the Pt/PC (89 mV), Pt/PC-H (79 mV), Pt/PC-H-500 (75 mV), Pt/PC-H-600 (65 mV), and Pt/PC-H-800 (73 mV) catalysts. Furthermore, the Pt/PC-H-700 catalyst also exhibited a smaller Tafel slope of 29.4 mV dec^−1^ than the Pt/PC (49.8 mV dec^−1^), Pt/PC-H (46.3 mV dec^−1^), Pt/PC-H-500 (44.8 mV dec^−1^), Pt/PC-H-600 (38.7 mV dec^−1^), and Pt/PC-H-800 (40.0 mV dec^−1^) catalysts (Figure 7c and Figure S9). These Tafel analyses indicated that the Pt/PC-H-700 catalyst had faster hydrogen evolution kinetics, meaning there was a smaller required overpotential for it to reach 10 mA cm^−2^. 

The HER reaction kinetics under polarization conditions and a constant overpotential of 90 mV were explored by EIS and Nyquist plots of the Pt/PC, Pt/PC-H, Pt/PC-H-500, Pt/PC-H-600, and Pt/PC-H-800 catalysts, which are given in Figure 7d. In general, the intersection of the semicircle with the real axis (Z_re_) in the Nyquist plot represents the equivalent series resistance (Rs), consisting of the electrolyte resistance, catalyst resistance, contact resistance of the electrode–catalyst interface, and interface resistance of the catalyst–electrolyte interface [61,62]. On the other hand, the diameter of the semicircle represents the charge transfer resistance (Rct) during the HER process [26,49]. Among the catalysts, Pt/PC-H-700 illustrated the smallest semicircle diameter when compared to Pt/PC, Pt/PC-H, Pt/PC-H-500, Pt/PC-H-600, and Pt/PC-H-800, indicating a favorable charge transfer resistance at the catalyst–electrolyte interface, conducive to the HER process. Accordingly, the Pt/PC-H-700 catalyst provided the smallest Rct value, demonstrating the best intrinsic activity among the investigated catalysts, consistent with the above LSV and Tafel analyses. From the fitted EIS results (Figure S10 and Table S4), the Rct value of the Pt/PC-H-700 (11.6 Ω) catalyst was lower than those of the Pt/PC (31.0 Ω), Pt/PC-H (25.1 Ω), Pt/PC-H-500 (22.3 Ω), Pt/PC-H-600 (17.2 Ω), and Pt/PC-H-800 (19.4 Ω) catalysts. Therefore, the Pt/PC-H-700 catalyst underwent superior charge transfer kinetics during the HER process. 

The HER durability features of the Pt/PC, Pt/PC-H, and Pt/PC-H-700 catalysts were evaluated through I-t scanning at a fixed overpotential of 110 mV. As illustrated in Figure 7e and Figure S11, the catalysts Pt/PC, Pt/PC-H, and Pt/PC-H-700 all showed drastic declines in their current densities during the initial HER stage, which was ascribed to the occupation of the active sites of the catalyst by the H_2_ bubbles generated during the initial HER process. Among the catalysts, Pt/PC-H-700 showed the highest initial current density of 47.0 mA cm^−2^ when compared to Pt/PC (26.4 mA cm^−2^) and Pt/PC-H (29.2 mA cm^−2^). After 10 h of the continuous HER process, Pt/PC-H-700 still exhibited a higher current density of 11.6 mA cm^−2^ than Pt/PC (3.6 mA cm^−2^) and Pt/PC-H (4.5 mA cm^−2^). Meanwhile, Pt/PC-H-700 displayed slightly slower attenuation in its current density with a 24.69% retained current density, superior to that of Pt/PC and Pt/Pt/PC-H, with current density delays of 13.66% and 15.34%, respectively. Thus, Pt/PC-H-700 possessed a better HER durability than Pt/PC and Pt/PC-H. The HER stability features of the Pt/PC, Pt/PC-H, and Pt/PC-H-700 catalysts were studied by ADT for 5000 continuous CV cycles. The LSV curves of the Pt/PC, Pt/PC-H, and Pt/PC-H-700 catalysts before and after the 5000 CV sweeps are presented in Figure S12. Obviously, Pt/PC-H-700 exhibited a smaller η_10_ decay of 10 mV than the Pt/PC (28 mV) and Pt/PC-H (10 mV), demonstrating a superior cycling stability. As depicted in Figure 7f and Figure S13, the V-t plot also showed that Pt/PC-H-700 displayed a slight change in its overpotential after 10 h at 10 mA cm^−2^ when compared to Pt/PC and Pt/PC-H. Therefore, the I-t, ADT, and V-t results all pointed to the excellent HER stability of Pt/PC-H-700 when compared to Pt/PC and Pt/PC-H, owing to the formation of thermally stable oxygen functional groups.

Overall, the structural analyses and HER electrocatalytic performances revealed the benefit of thermally stable oxygen functional groups, such as ether groups and carbonyl groups, on carbon supports in the dispersion and anchoring of Pt nanoparticles, as well as the enhancement of the interfacial interaction between the carbon supports and catalysts, leading to improved HER catalytic performances of the catalysts. 

## 4. Conclusions

The role of oxygen functionalization via a HCl treatment of porous carbon supports on the HER performance of Pt/C catalysts was successfully clarified. After the HCl treatment followed by a heat treatment, the structural characterizations showed negligible changes in the morphologies, specific surface areas, pore volumes, and microcrystallines of the supports. However, the HCl treatment introduced enriched hydroxyl and carboxyl groups, while the further heat treatment transformed the functional groups into thermally stable carbonyl and ether groups. As a result, the optimized Pt/PC-H-700 catalyst exhibited an elevated HER activity with a low overpotential of 50 mV at 10 mA cm^−2^, better than those of the Pt/PC (89 mV) and Pt/PC-H (79 mV) catalysts. According to V-t, I-t, and ADT analyses, the Pt/PC-H-700 catalyst also displayed a better HER stability than the Pt/PC and Pt/PC-H catalysts. In sum, the interaction between the Pt nanoparticles and porous carbon supports could be improved by optimizing the surface chemistry properties of the carbon supports. In other words, the surface properties of porous carbon supports may be modified by a HCl treatment followed by an appropriate heat treatment, which is suitable for the rational optimization and regulation of the oxygen functional group species on porous carbon supports for enhanced HER performances.

## Figures and Tables

**Table 1:** Textural parameters of PC, PC-H, PC-H-500, PC-H-600, PC-H-700, and PC-H-800 supports.

Supports	^a^ S_BET_ (m^2^·g^−1^)	^b^ S_QSDFT_ (m^2^·g^−1^)	^c^ S_micro_ (m^2^·g^−1^)	^d^ S_external_ (m^2^·g^−1^)	^e^ V_total_ (cm^3^·g^−1^)	^f^ V_QSDFT_ (cm^3^·g^−1^)	^g^ V_micro_ (cm^3^·g^−1^)	^h^ V_external_ (cm^3^·g^−1^)
PC	1751	1605	1501	104	0.94	0.90	0.73	0.17
PC-H	1796	1636	1517	119	0.99	0.95	0.75	0.20
PC-H-500	1857	1663	1570	93	0.97	0.92	0.77	0.15
PC-H-600	1809	1640	1523	117	0.99	0.95	0.76	0.19
PC-H-700	1888	1650	1538	113	1.00	0.96	0.78	0.18
PC-H-800	1845	1647	1546	101	0.96	0.92	0.76	0.16

^a^ BET-specific surface area (S_BET_); ^b^ QSDFT specific surface area (S_QSDFT_); ^c^ Micropore-specific surface area based on QSDFT (S_micro_); ^d^ S_external_ = S_QSDFT_ − S_micro_; ^e^ Single-point total pore volume at P/P_0_ = 0.99 (V_total_); ^f^ Total pore volume based on QSDFT (V_QSDFT_); ^g^ Micropore volume based on QSDFT (V_micro_); and ^h^ V_external_ = V_QSDFT_ − V_micro_.

## Data Availability

The data presented in this study are available in this article and its Supplementary Materials.

## Supplementary Materials

The following supporting information can be downloaded at: www.mdpi.com/article/10.3390/nano13081415/s1, Figure S1: Cumulative pore volume of PC, PC-H, PC-H-500, PC-H-600, PC-H-700, and PC-H-800 supports; Figure S2: Fitted Raman spectra of the PC (a), PC-H (b), PC-H-500 (c), PC-H-600 (d), PC-H-700 (e), and PC-H-800 (f) supports; Figure S3: XPS surveys of PC, PC-H, PC-H-500, PC-H-600, PC-H-700, and PC-H-800; Figure S4: High-resolution C 1s spectra of PC (a), PC-H (b), PC-H-500 (c), PC-H-600 (d), PC-H-700 (e), and PC-H-800 (f) supports; Figure S5: XPS surveys of Pt/PC, Pt/PC-H, and Pt/PC-H-700 catalysts; Figure S6: High-resolution C 1s (a–c) and O 1s (d-f) spectra of Pt/PC, Pt/PC-H, and Pt/PC-H-700 catalysts, respectively; Figure S7: SEM images (a,b) and corresponding EDX mappings (c-f) of Pt/PC-H-700 catalyst; Figure S8: TEM images of Pt/PC (a) and Pt/PC-H-700 (b) catalysts; Figure S9: Tafel slope values of Pt/PC, Pt/PC-H, Pt/PC-H-500, Pt/PC-H-600, Pt/PC-H-700, and Pt/PC-H-800 catalysts; Figure S10: Experimental and simulated Nyquist plots of Pt/PC, Pt/PC-H, Pt/PC-H-500, Pt/PC-H-600, Pt/PC-H-700, and Pt/PC-H-800 catalysts at −90 mV (inset shows the equivalent circuit model); Figure S11: Current densities of Pt/PC, Pt/PC-H, and Pt/PC-H-700 catalysts in I-t curves at initial, 2 h, 4 h, 6 h, 8 h, and 10 h; Figure S12: LSV curves of Pt/PC (b), Pt/PC-H (c), and Pt/PC-H-700 (d) catalysts before and after 5000 CV cycles; Figure S13: Overpotentials of Pt/PC, Pt/PC-H, and Pt/PC-H-700 catalysts in V-t curves at 1 h, 2 h, 4 h, 6 h, 8 h, and 10 h; Table S1: The content percentages from the decomposed C 1s spectra of Pt/PC, Pt/PC-H, and Pt/PC-H-700 catalysts; Table S2: The content percentages from the decomposed O 1s spectra of Pt/PC, Pt/PC-H, and Pt/PC-H-700 catalysts; Table S3: The content percentages from the decomposed Pt 4f spectra of Pt/PC, Pt/PC-H, and Pt/PC-H-700 catalysts; Table S4: EIS fitting results of Pt/PC, Pt/PC-H, Pt/PC-H-500, Pt/PC-H-600, Pt/PC-H-700, and Pt/PC-H-800 catalysts.
