# Supplementary material for: The Enhancing Effect of Stable Oxygen Functional Groups on Porous-Carbon-Supported Pt Catalysts for Alkaline Hydrogen Evolution
Source: Nanomaterials (Basel). 2023 Apr 20;13(8):1415. doi: 10.3390/nano13081415 (PMC10145733; doi:10.3390/nano13081415)
Supplement: Supplementary file 1 [file nanomaterials-13-01415-s001.zip › nanomaterials-2317419-supplementary.pdf]

The Enhancing Effect of Stable Oxygen Functional Groups on Porous-Carbon-Supported Pt Catalysts for Alkaline Hydrogen Evolution

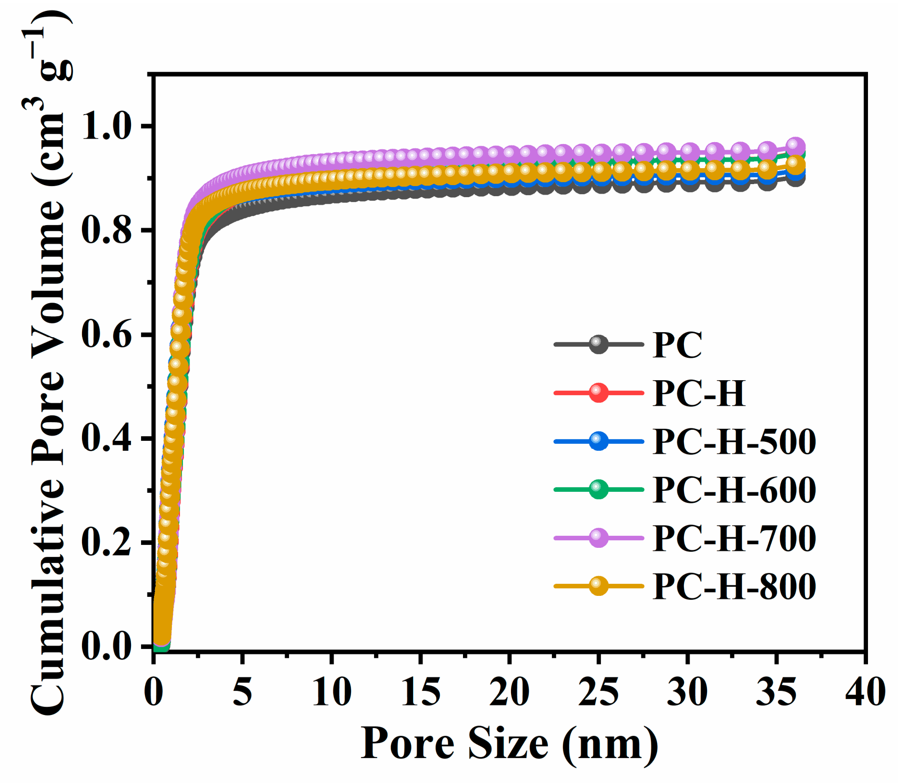

Figure S1. Cumulative pore volume of PC, PC-H, PC-H-500, PC-H-600, PC-H-700, and PC-H-800 supports.

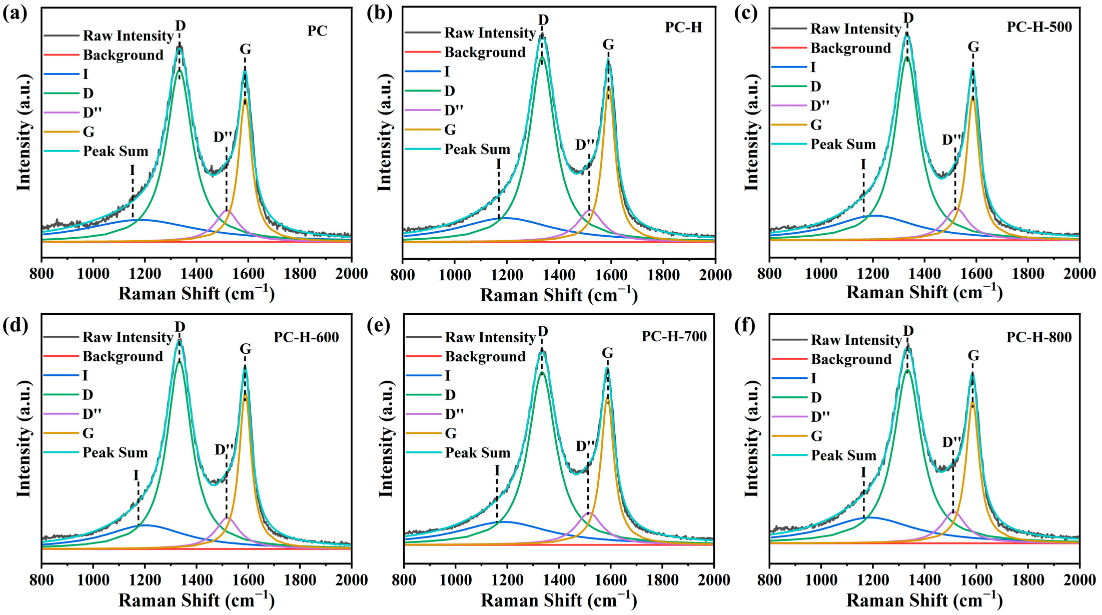

Figure S2. Fitted Raman spectra of the PC (a), PC-H (b), PC-H-500 (c), PC-H-600 (d), PC-H-700 (e), and PC-H-800 (f) supports.

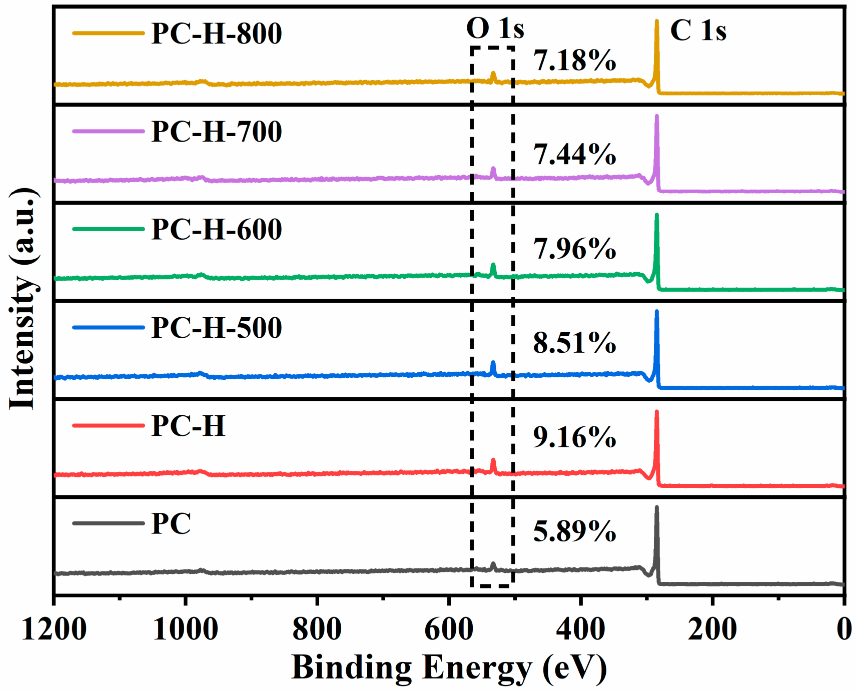

Figure S3. XPS surveys of PC, PC-H, PC-H-500, PC-H-600, PC-H-700, and PC-H-800.

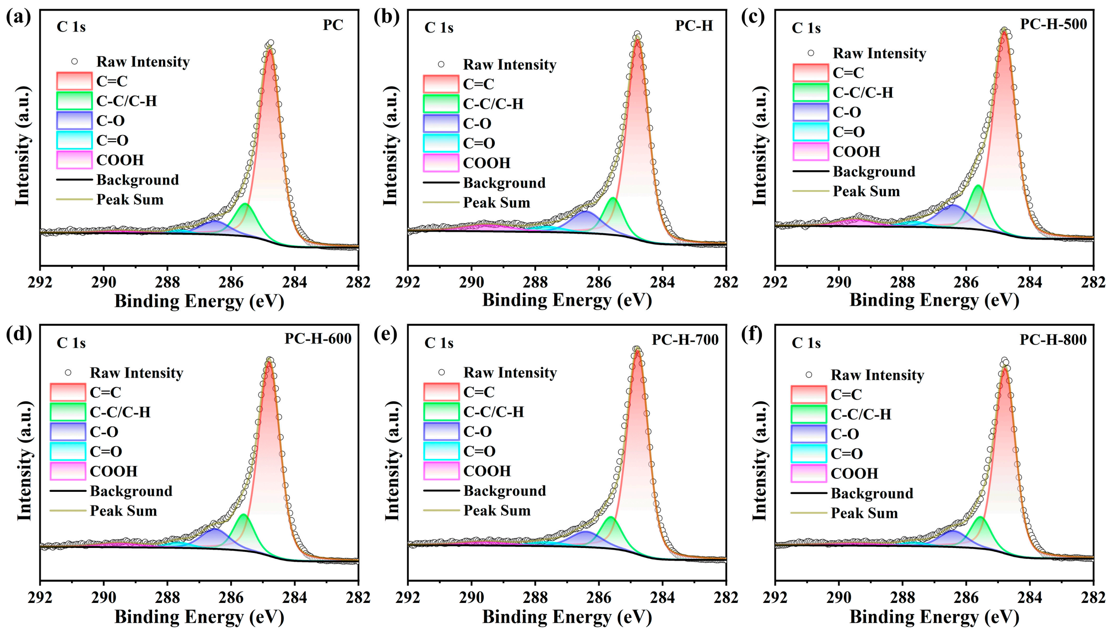

Figure S4. High-resolution C 1s spectra of PC (a), PC-H (b), PC-H-500 (c), PC-H-600 (d), PC-H-700 (e), and PC-H-800 (f) supports.

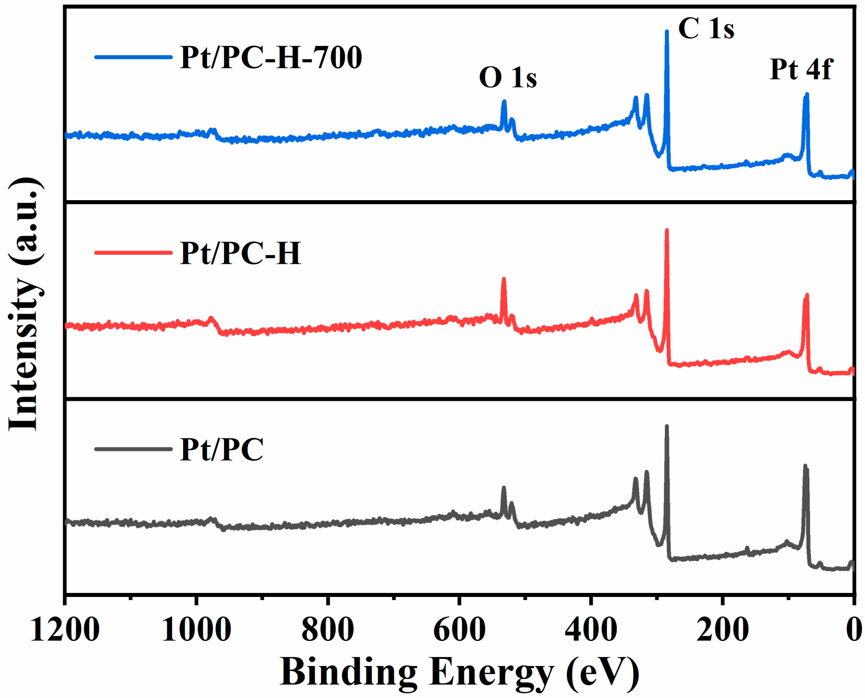

Figure S5. XPS surveys of Pt/PC, Pt/PC-H, and Pt/PC-H-700 catalysts.

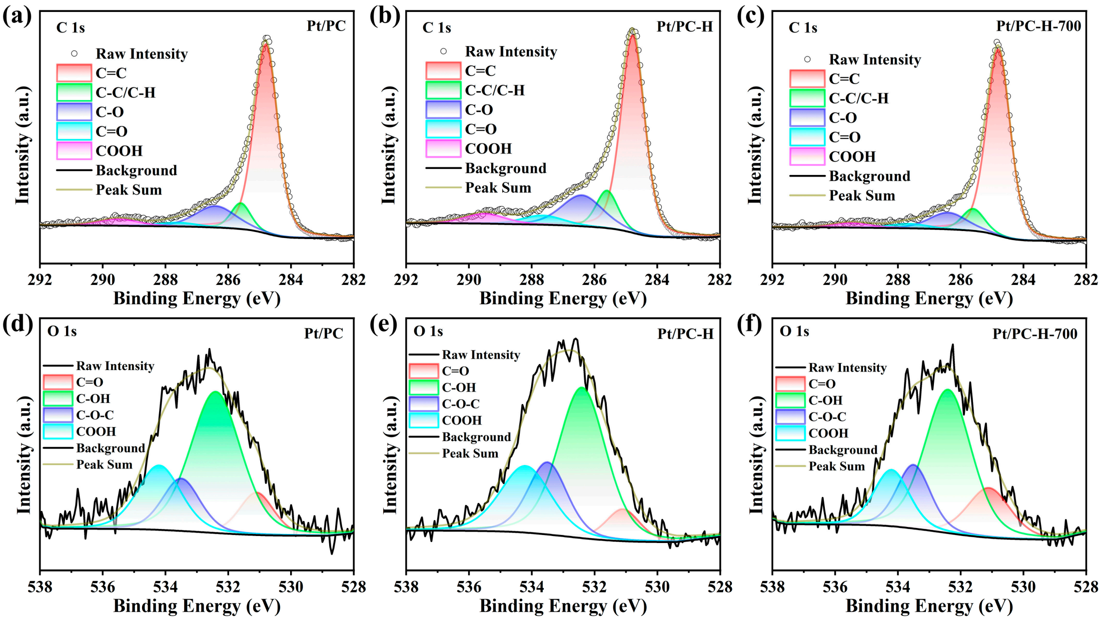

Figure S6. High-resolution C 1s (a-c) and O 1s (d-f) spectra of Pt/PC, Pt/PC-H, and Pt/PC-H-700 catalysts, respectively.

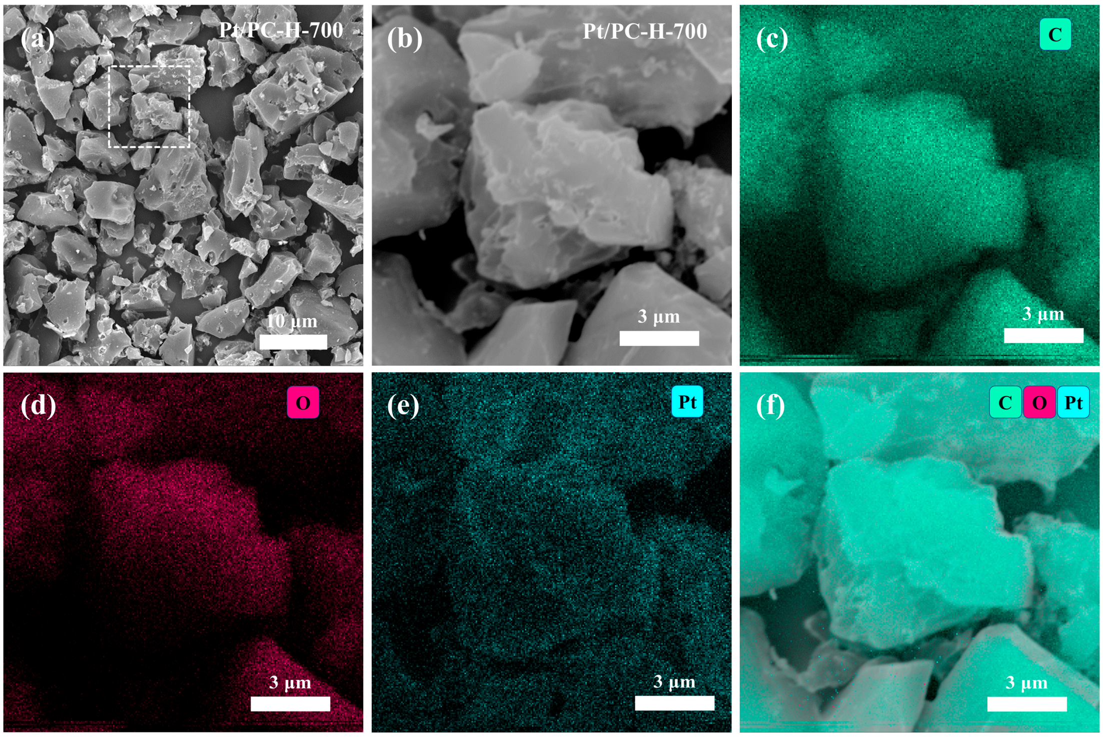

Figure S7. SEM images (a, b) and corresponding EDX mappings (c-f) of Pt/PC-H-700 catalyst.

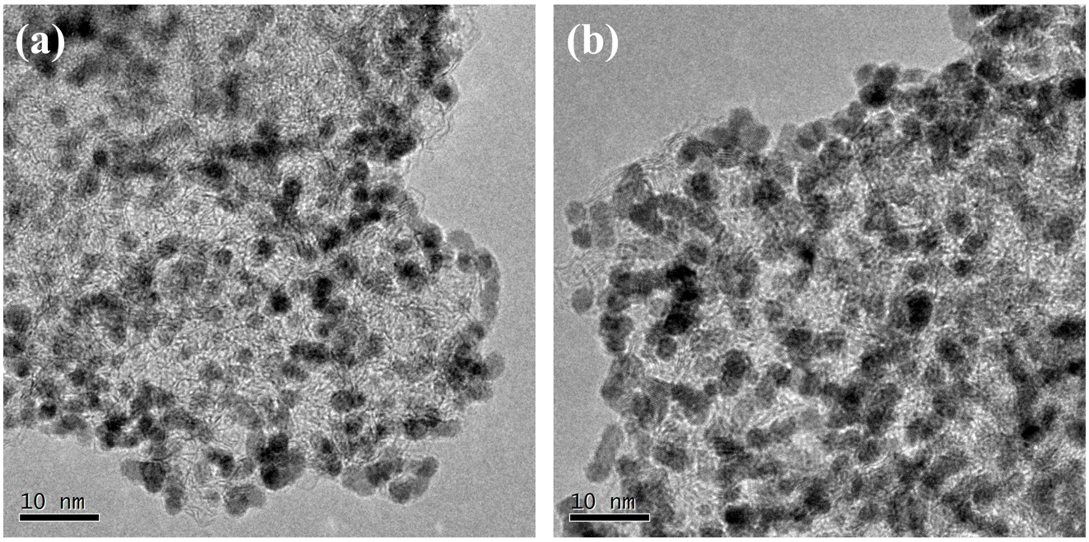

Figure S8. TEM images of Pt/PC (a) and Pt/PC-H-700 (b) catalysts.

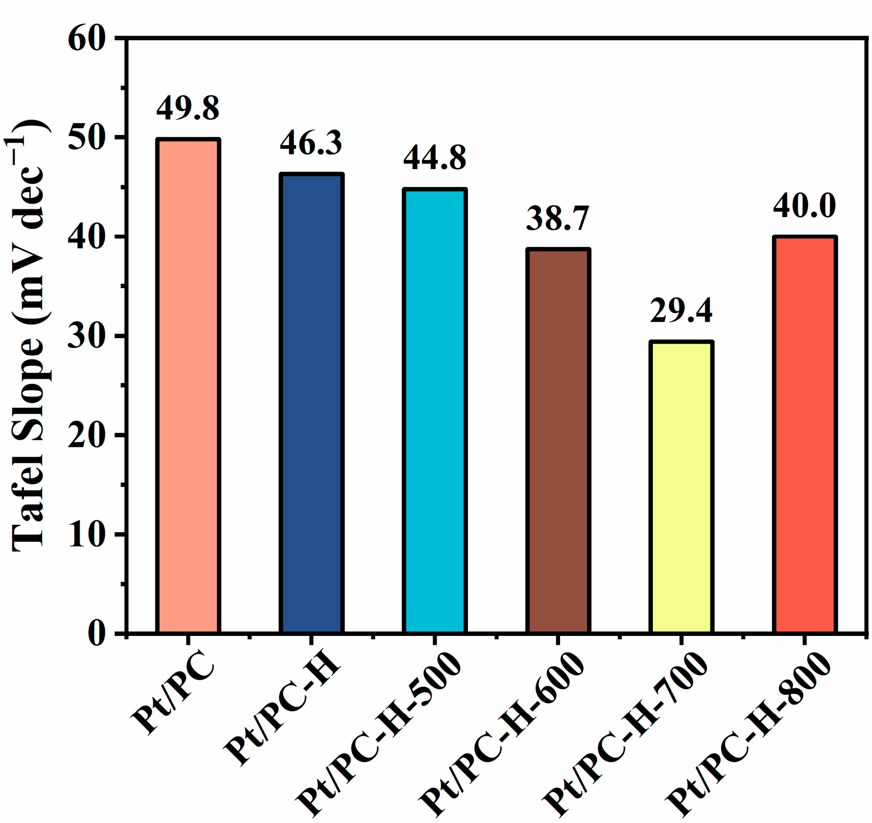

Figure S9. Tafel slope values of Pt/PC, Pt/PC-H, Pt/PC-H-500, Pt/PC-H-600, Pt/PC-H-700, and Pt/PC-H-800 catalysts.

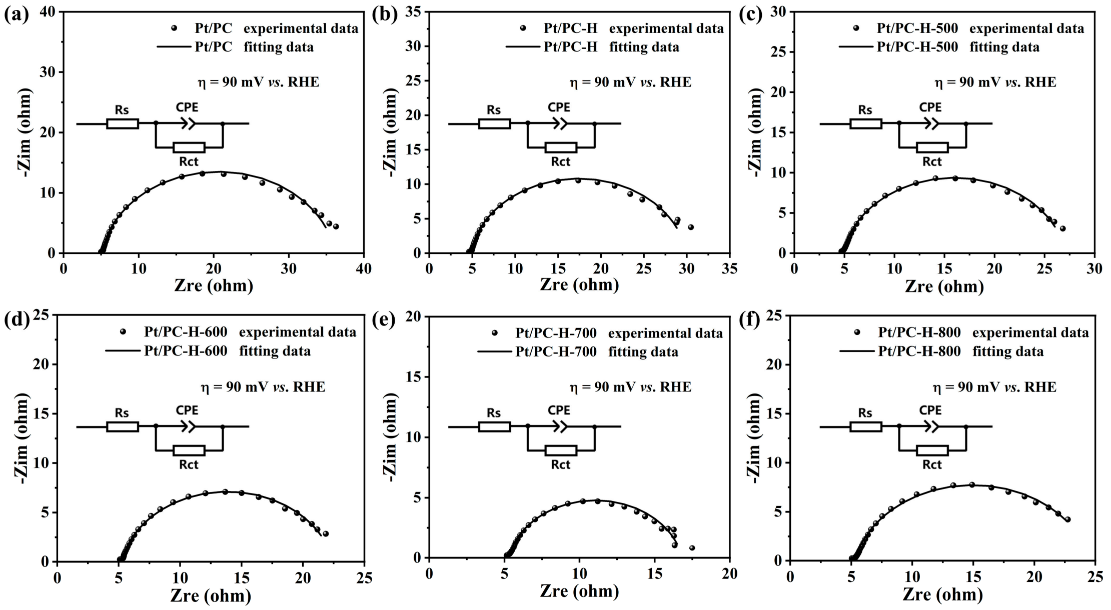

Figure S10. Experimental and simulated Nyquist plots of Pt/PC, Pt/PC-H, Pt/PC-H-500, Pt/PC-H-600, Pt/PC-H-700, and Pt/PC-H-800 catalysts at -90 mV (inset shows the equivalent circuit model).

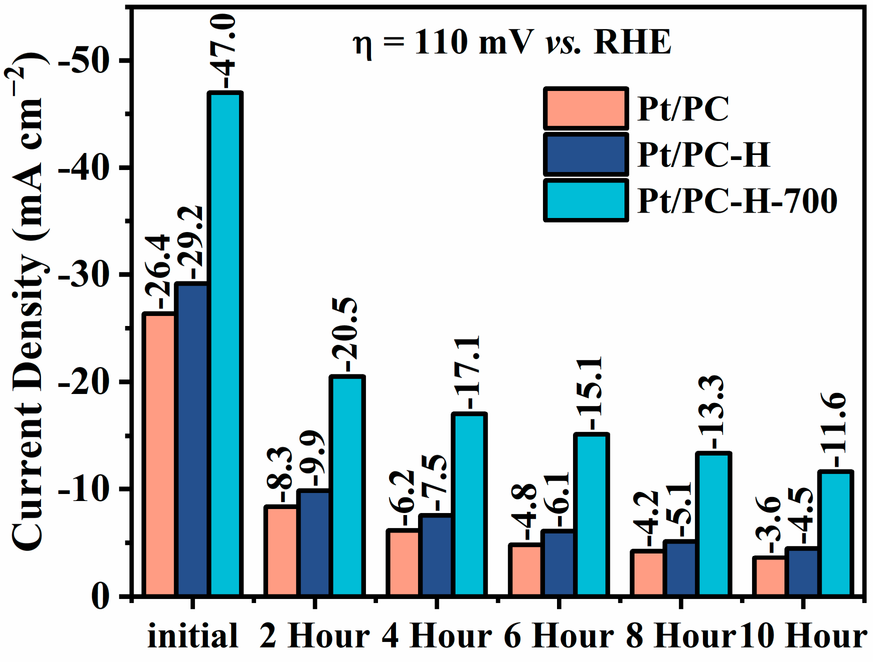

Figure S11. Current densities of Pt/PC, Pt/PC-H, and Pt/PC-H-700 catalysts in I-t curves at initial, 2 h, 4 h, 6 h, 8 h, and 10 h.

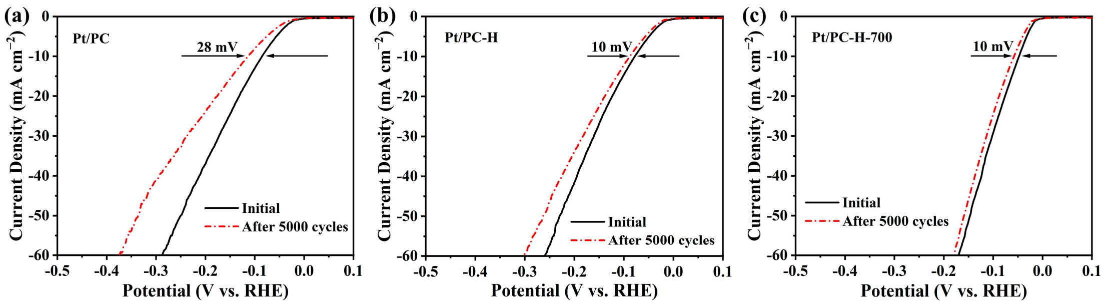

Figure S12. LSV curves of Pt/PC (b), Pt/PC-H (c), and Pt/PC-H-700 (d) catalysts before and after 5, 000 CV cycles.

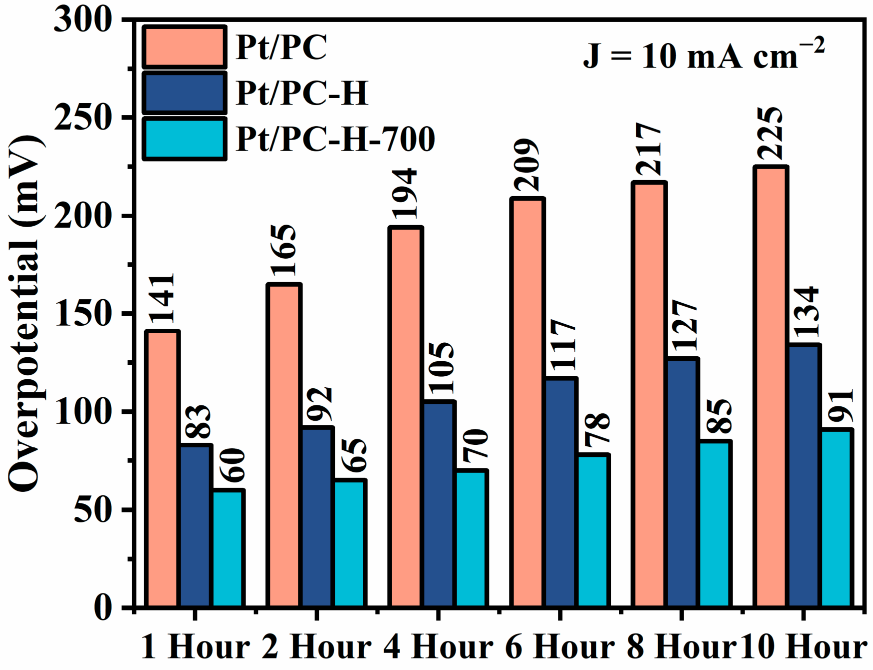

Figure S13. Overpotentials of Pt/PC, Pt/PC-H, and Pt/PC-H-700 catalysts in V-t curves at 1 h, 2 h, 4 h, 6 h, 8 h, and 10 h.

Table S1. The content percentages from the decomposed C 1s spectra of Pt/PC, Pt/PC-H, and Pt/PC-H-700 catalysts.

Catalysts	C=C	C-C/C-H	C-O	C=O	COOH
Pt/PC	71.4%	8.1%	13.7%	2.5%	4.3%
Pt/PC-H	61.8%	10.7%	16.9%	5.1%	5.5%
Pt/PC-H-700	75.0%	8.4%	10.7%	2.9%	3.1%

Table S2. The content percentages from the decomposed O 1s spectra of Pt/PC, Pt/PC-H, and Pt/PC-H-700 catalysts.

Catalysts	C=O	C-OH	C-O-C	COOH
Pt/PC	10.9%	53.1%	14.9%	21.1%
Pt/PC-H	7.4%	49.9%	19.3%	23.5%
Pt/PC-H-700	16.4%	51.4%	16.7%	15.5%

Table S3. The content percentages from the decomposed Pt 4f spectra of Pt/PC, Pt/PC-H, and Pt/PC-H-700 catalysts.

Catalysts	Pt^0 $4f_{7/2}$	Pt^0 $4f_{5/2}$	Pt^{2+} $4f_{7/2}$	Pt^{2+} $4f_{5/2}$	Pt^{4+} $4f_{7/2}$	Pt^{4+} $4f_{5/2}$
Pt/PC	19.3%	15.2%	21.7%	17.1%	14.9%	11.7%
Pt/PC-H	17.4%	13.7%	23.2%	18.3%	15.4%	12.1%
Pt/PC-H-700	19.2%	15.1%	22.6%	17.8%	14.1%	11.1%

Table S4. EIS fitting results of Pt/PC, Pt/PC-H, Pt/PC-H-500, Pt/PC-H-600, Pt/PC-H-700, and Pt/PC-H-800 catalysts.

Catalysts	Rs (Ohm, Ω)	Rct (Ohm, Ω)	Fitting error (χ^2)
Pt/PC	5.1	31.0	5.21E-04
Pt/PC-H	4.8	25.1	5.37E-04
Pt/PC-H-500	4.8	22.3	5.38E-04
Pt/PC-H-600	5.2	17.2	3.44E-04
Pt/PC-H-700	5.2	11.6	4.03E-04
Pt/PC-H-800	5.2	19.4	5.77E-04
